# Supplementary material for: Cryo-EM reveals an extrahelical allosteric binding site at the M5 mAChR
Source: Nat Commun. 2025 Jul 31;16:7046. doi: 10.1038/s41467-025-62212-z (PMC12314052; doi:10.1038/s41467-025-62212-z)
Supplement: Supplementary file 1 — Supplementary Information [file 41467_2025_62212_MOESM1_ESM.pdf]

Supplementary Information for

Cryo-EM reveals an extrahelical allosteric binding site at the M_5 mAChR

Wessel A. C. Burger[1,2,*], Jesse I. Mobbs[1,2,*], Bhavika Rana[1,2,*], Jinan Wang[3,*], Keya Joshi[3], Patrick R. Gentry[1], Mahmuda Yeasmin[1,2], Hariprasad Venugopal[4], Aaron M. Bender[5], Craig W. Lindsley[5], Yinglong Miao[3], Arthur Christopoulos[1,2,6], Celine Valant[1,2], David M. Thal[1,2].

Affiliations

1. Drug Discovery Biology, Monash Institute of Pharmaceutical Sciences, Monash University, Parkville, Victoria, Australia.
2. Australian Research Council Centre for Cryo-Electron Microscopy of Membrane Proteins, Monash Institute of Pharmaceutical Sciences, Monash University, Parkville, Victoria, Australia.
3. Computational Medicine Program and Department of Pharmacology, University of North Carolina−Chapel Hill, Chapel Hill, North Carolina 27599, United States.
4. Ramaciotti Centre for Cryo-Electron Microscopy, Monash University, Clayton, Victoria, Australia.
5. Department of Pharmacology, Warren Center for Neuroscience Drug Discovery and Department of Chemistry, Vanderbilt University, Nashville, United States
6. Neuromedicines Discovery Centre, Monash University, Parkville, Australia.

*These authors contributed equally: Wessel A. C. Burger, Jesse I. Mobbs, Bhavika Rana, Jinan Wang.

E-mail: Yinglong Miao: Yinglong_Miao@med.unc.edu; Arthur Christopoulos: arthur.christopoulos@monash.edu; Celine Valant: celine.valant@monash.edu; David M. Thal: david.thal@monash.edu

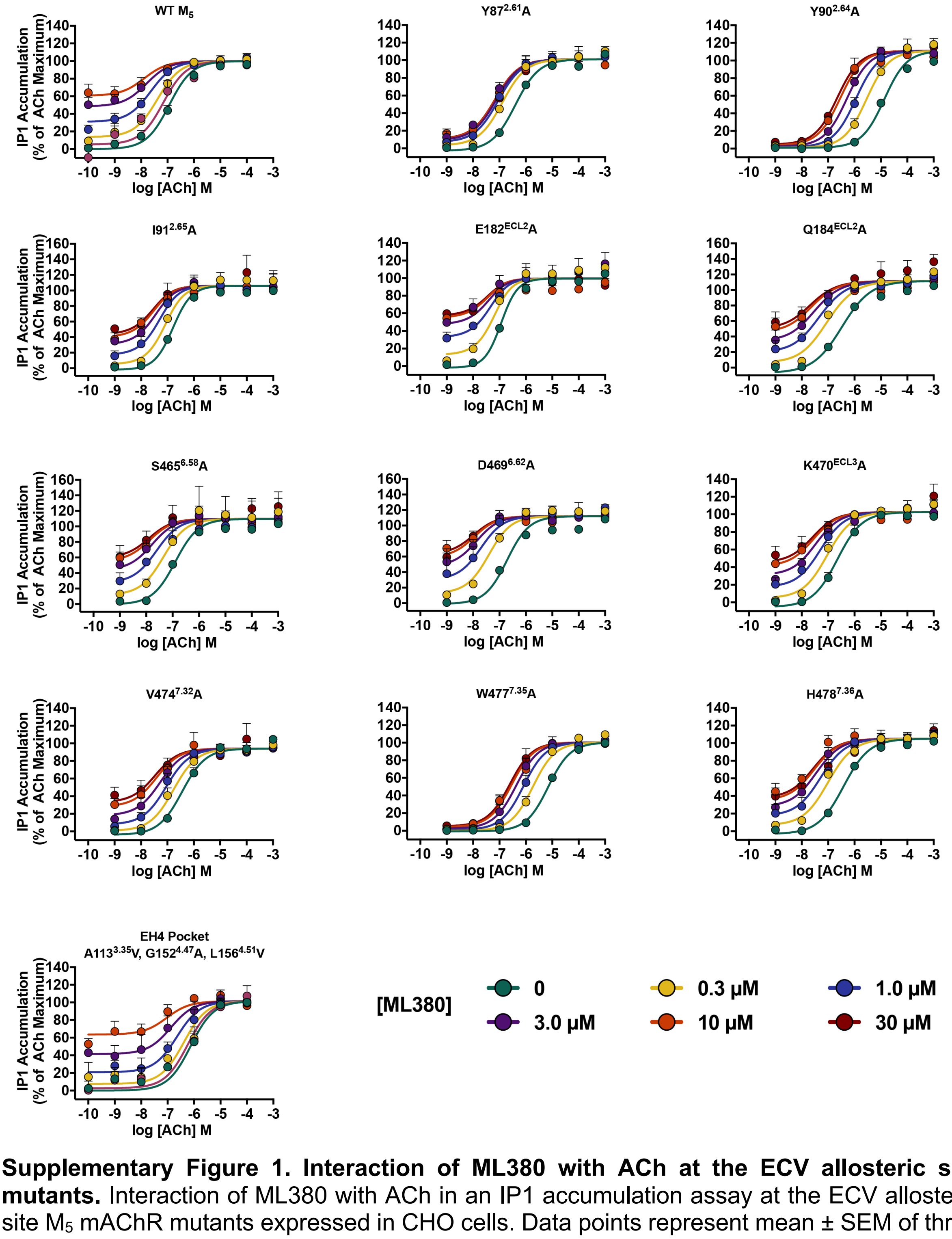

Supplementary Figure 1. Interaction of ML380 with ACh at the ECV allosteric site mutants. Interaction of ML380 with ACh in an IP1 accumulation assay at the ECV allosteric site M_5 mAChR mutants expressed in CHO cells. Data points represent mean ± SEM of three individual experiments performed in duplicate (WT M_5, n=7). An operational model of allosterism was fit to the data. Parameters obtained are listed in Supplementary Table 1. Graphs for WT M_5 mAChR and EH4 pocket were reproduced from the main Figure 1a.

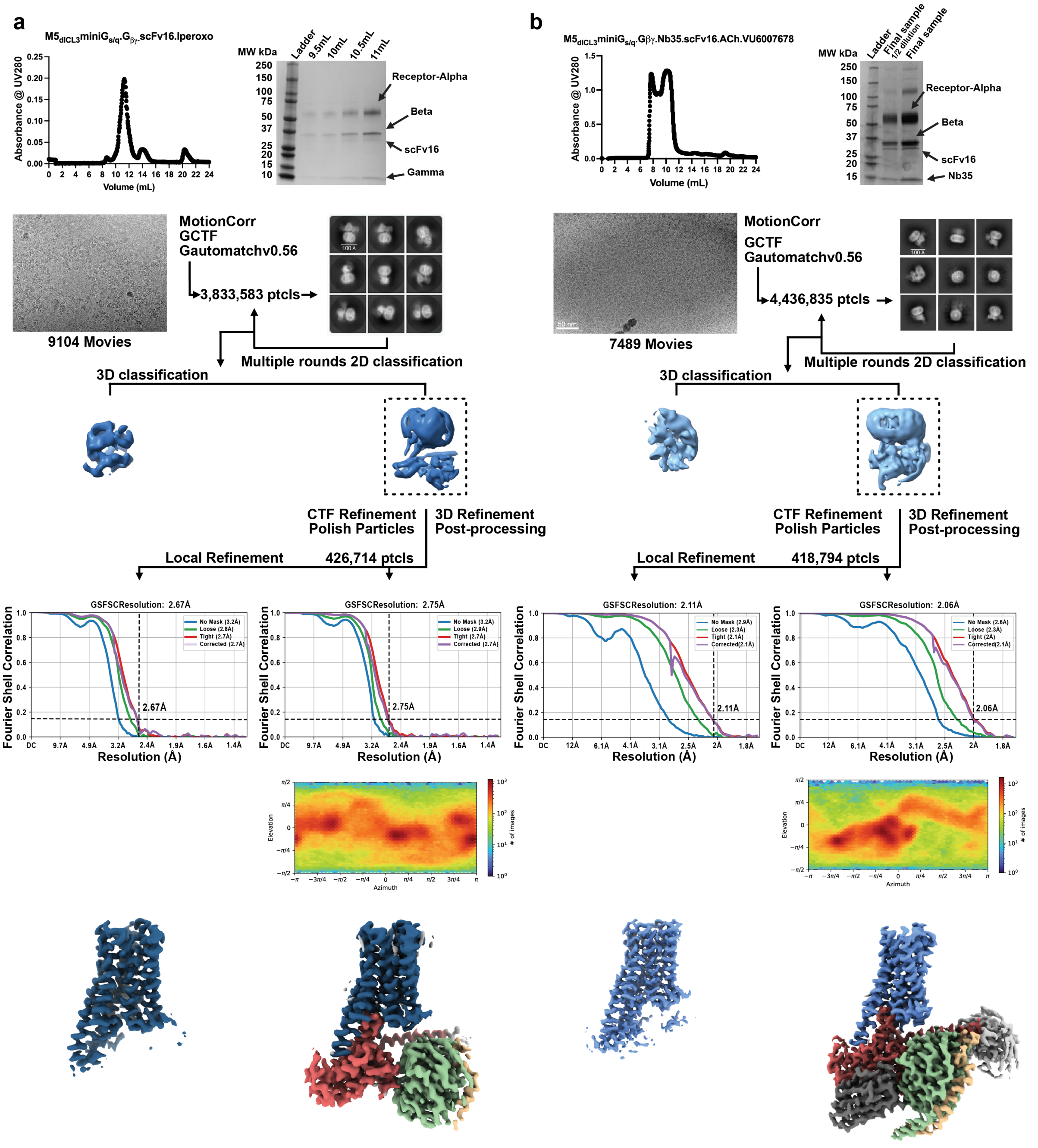

Supplementary Figure 2. Cryo-EM data processing for the iperoxo-bound M_5 mAChR-mGα_q and ACh-VU6007678-bound M_5 mAChR-mGα_q complexes. Flow chart for cryo-EM analysis as described in methods leading to final cryo-EM density maps for **(a)** the iperoxo-bound M_5 mAChR-mGα_q complex and **(b)** ACh-VU6007678-bound M_5 mAChR-mGα_q complex.

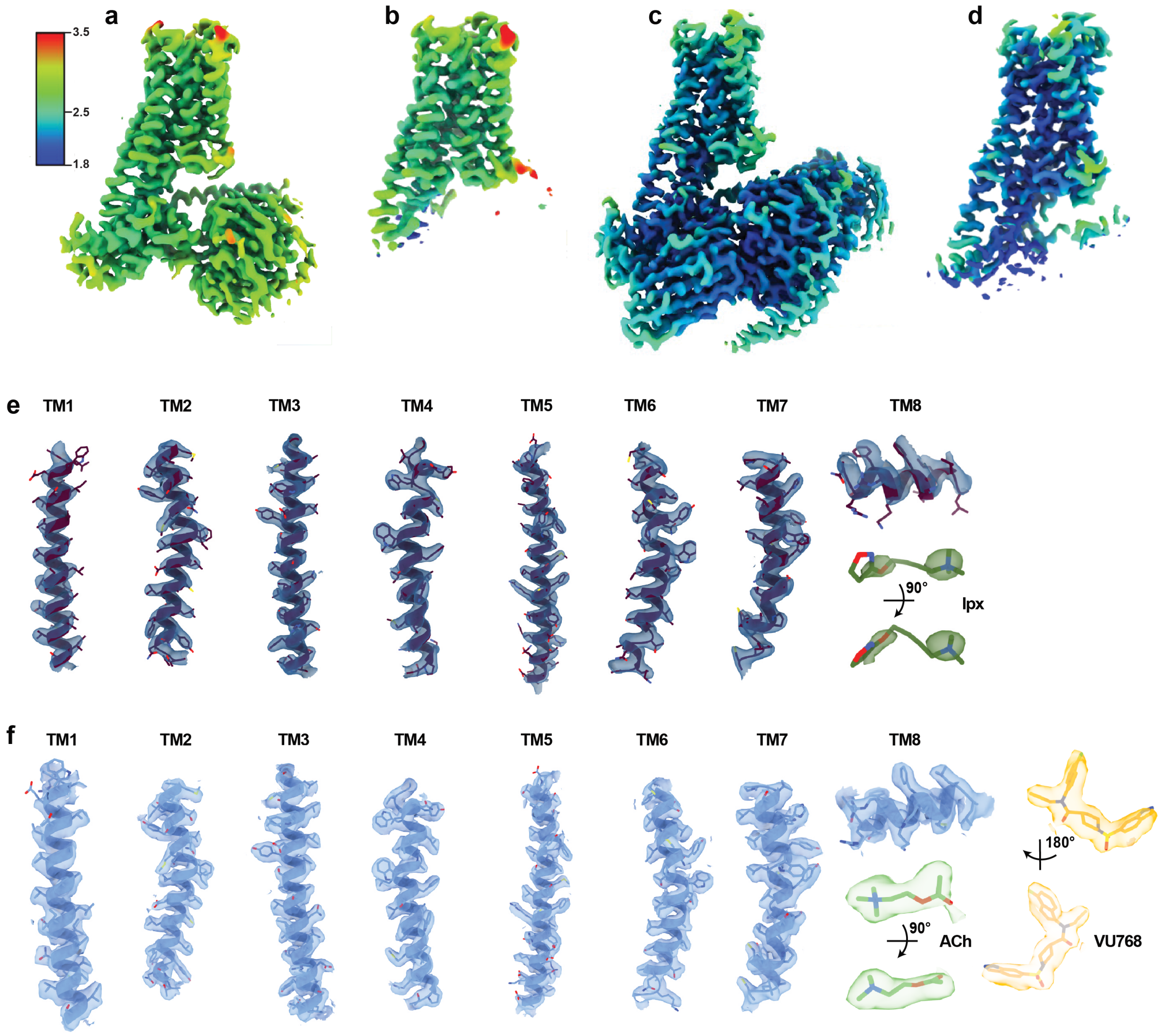

Supplementary Figure 3. Cryo-EM density maps. Local resolution map of the **(a)** complex, **(b)** receptor maps for the iperoxo-bound M_5 mAChR-mGα_q structure and **(c)** complex, **(d)** receptor maps for the ACh-VU6007678-bound M_5 mAChR-mGα_q structure calculated in RELION 3.1. **(e-f)** Shown as cartoons, the transmembrane domains of the M_5 mAChR and the α5 of G_q are modelled into the receptor-focused cryo-EM maps, which are shown as a transparent surface contoured at 0.332 for (e) and 0.344 for (f).

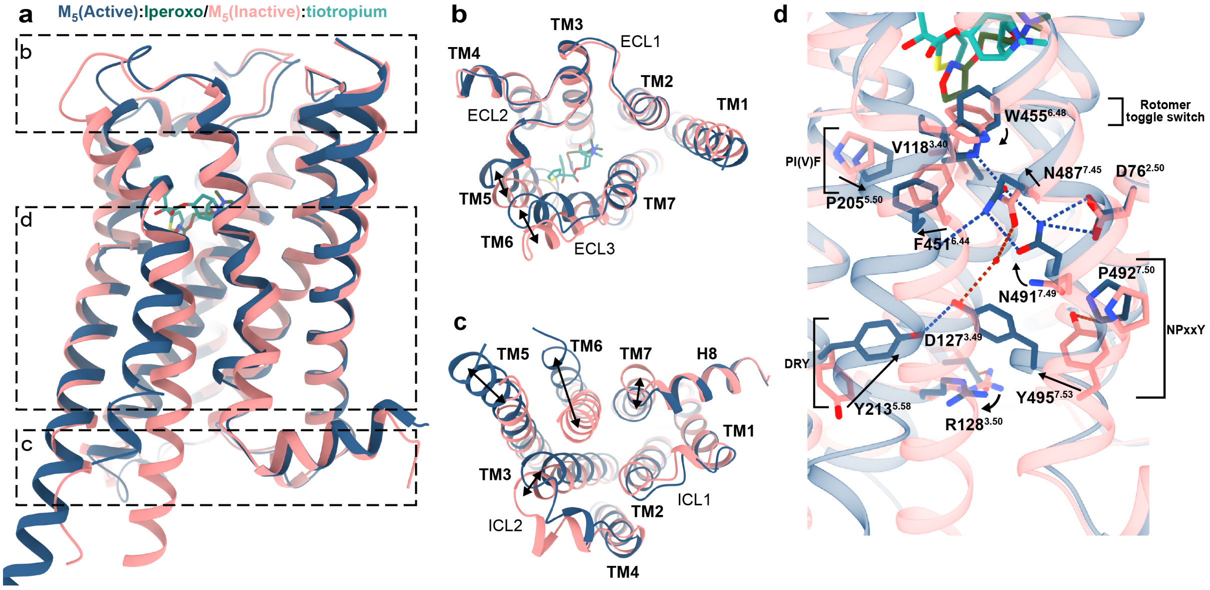

Supplementary Figure 4. Comparison of active and inactive M$_5$ mAChR. (a) Overlay of active, iperoxo-bound M$_5$ mAChR (shown in dark blue) with inactive, tiotropium-bound M$_5$ mAChR (shown in salmon, PDB: 6OL9). **(b)** Extracellular view comparing ECLs and TM regions. Arrows indicate movement between inactive and active states. **(c)** Intracellular view (G protein removed) comparing ICLs and TM regions. Arrows indicate movement between inactive and active states. **(d)** Key receptor activation motifs for class A GPCRs. Arrows indicate movement of residues between inactive and active states. Red dashed lines indicate hydrogen bonds in the inactive structure. Blue dashed lines indicate hydrogen bonds in the active structure.

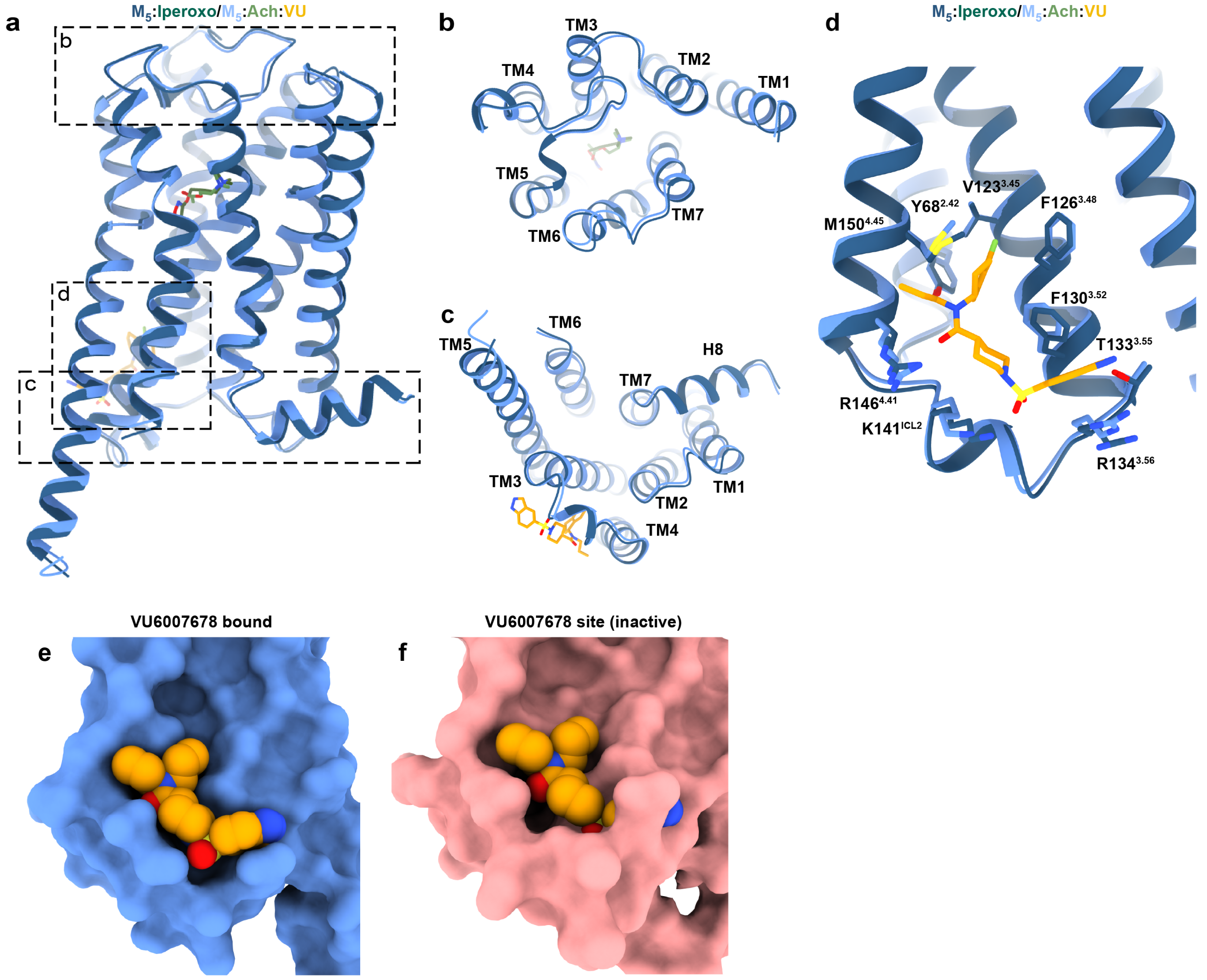

Supplementary Figure 5. Comparison of iperoxo bound to ACh-VU6007678 bound M_5 mAChR. (a) Overlay of iperoxo-bound M_5 mAChR (shown in dark blue) with ACh-VU6007678-bound M_5 mAChR (shown in light blue). Iperoxo is shown in dark green sticks, ACh in light green sticks and VU6007678 in orange sticks. **(b)** Extracellular view comparing ECLs and TM regions. **(c)** Intracellular view (G protein removed) comparing ICLs and TM regions. **(d)** Comparison of the allosteric binding site at the ACh-VU6007678 bound M_5 mAChR to the iperoxo bound M_5 mAChR. Key residues are shown as sticks, iperoxo-bound M_5 mAChR in dark blue, ACh-VU6007678 M_5 mAChR bound in light blue, and VU6007678 is represented as orange sticks. **(e-f)** Spherical representation of the VU6007678 allosteric binding site at (e) ACh-VU6007678 bound M_5 mAChR and (f) inactive, tiotropium-bound M_5 mAChR (PDB:6OL9).

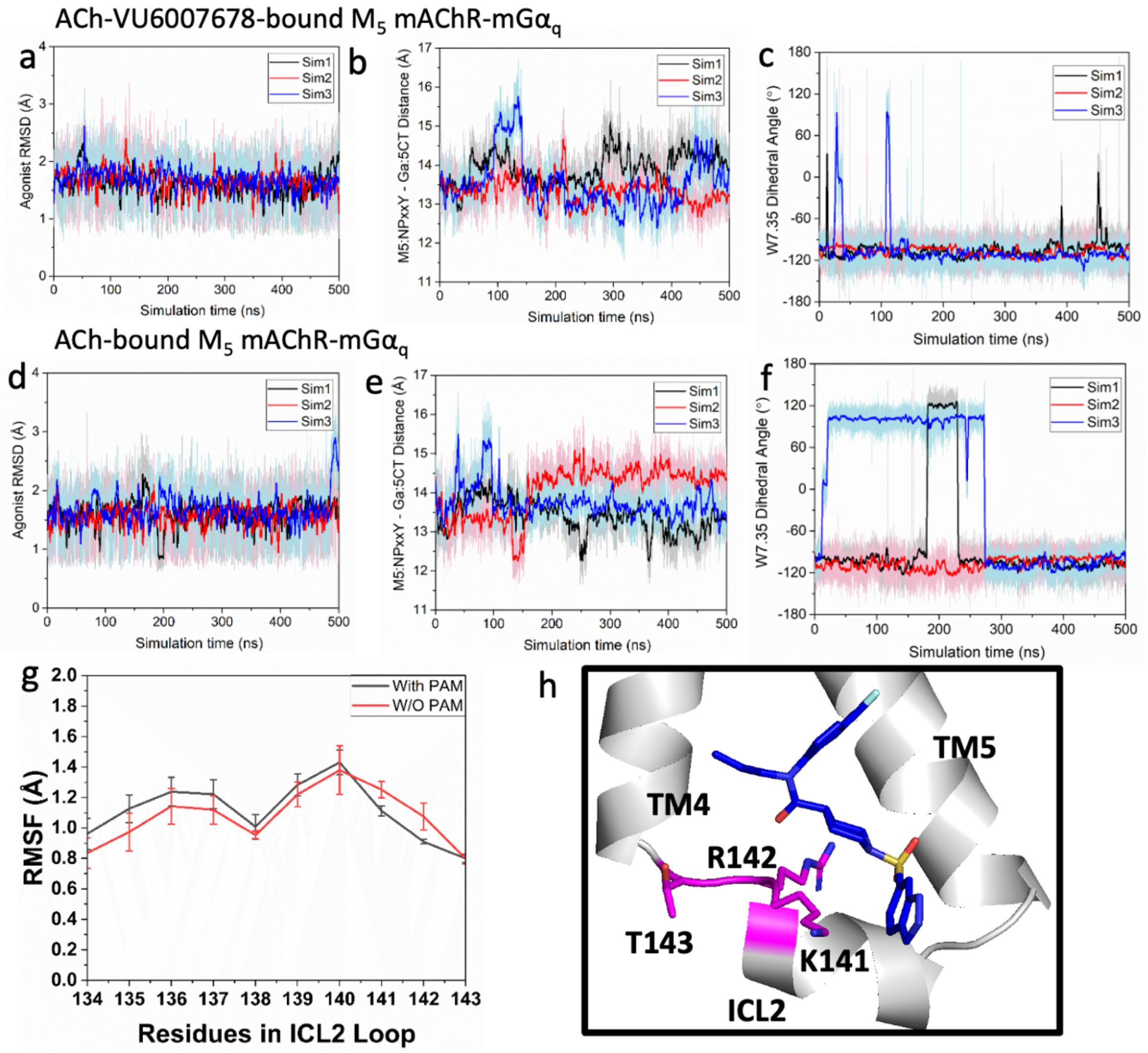

Supplementary Figure 6. Comparison of GaMD simulations with ACh-VU6007678- and ACh-bound M_5 mAChR-mGα_q. Time courses of distances (Å) from Gaussian accelerated molecular dynamics (GaMD) simulations performed on (**a-c**) the ACh-VU6007678-bound M_5 mAChR-mGα_q structure and (**d-f**) with VU6007678 removed. (**g**) Comparison of the root mean square fluctuations (RMSF, mean ± SEM) of residues in the ICL2 loop. (**h**) Residues in purple show reduced RMSF values in the presence of VU6007678 (coloured in blue) and stabilise an additional one residue turn of ICL2. GaMD simulations were performed from three separate replicates, as indicated by different-coloured traces.

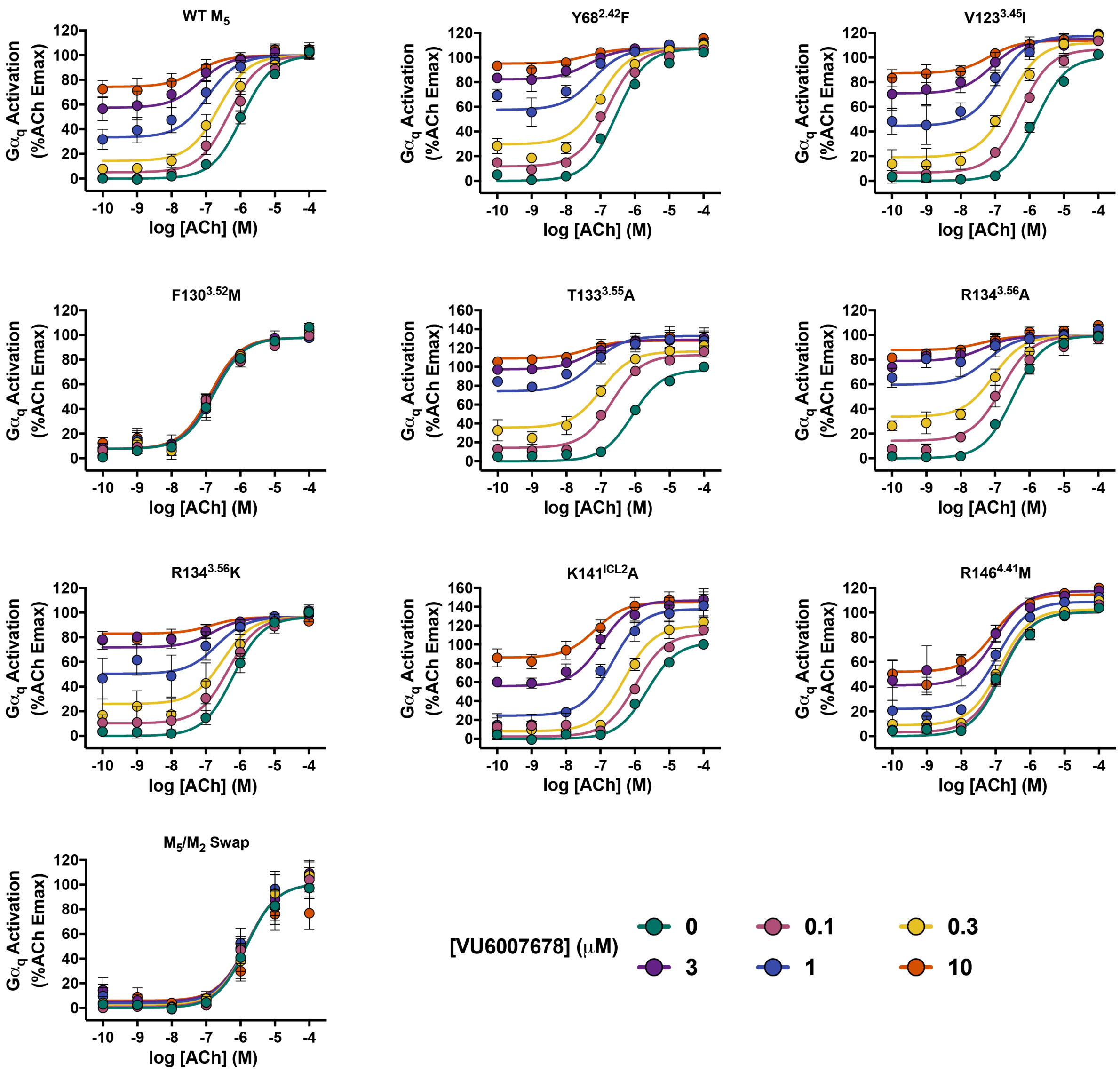

Supplementary Figure 7. Functional interaction of VU6007678 with ACh at allosteric site mutants. Interaction of VU6007678 with ACh in a Trupath G$_q$ activation assay at the allosteric site mutant M$_5$ mAChRs expressed in CHO cells. Grouped data points represent mean ± SEM of three to eight individual experiments performed in duplicate. WT M$_5$ mAChR *n* = 8, M$_5$/M$_2$ swap, F130$^{3.52}$M, T133$^{3.55}$A, R134$^{3.56}$A, R134$^{3.56}$K mutants *n* = 3, Y68$^{2.42}$F, V123$^{3.45}$I, K141$^{34.56}$A, R146$^{4.41}$M mutants *n* = 4. An operational model of allosterism was fit to the data. Pharmacology parameters were shared across experiments to yield a single best estimate of each parameter and its associated standard error, as derived from the nonlinear regression algorithm. This global pooled analysis approach ensured model convergence in all instances. Parameters obtained are listed in Supplementary Table 4. Graphs for WT M$_5$ mAChR and M$_5$/M$_2$ swap were reproduced from the main Figure 5 b-c.

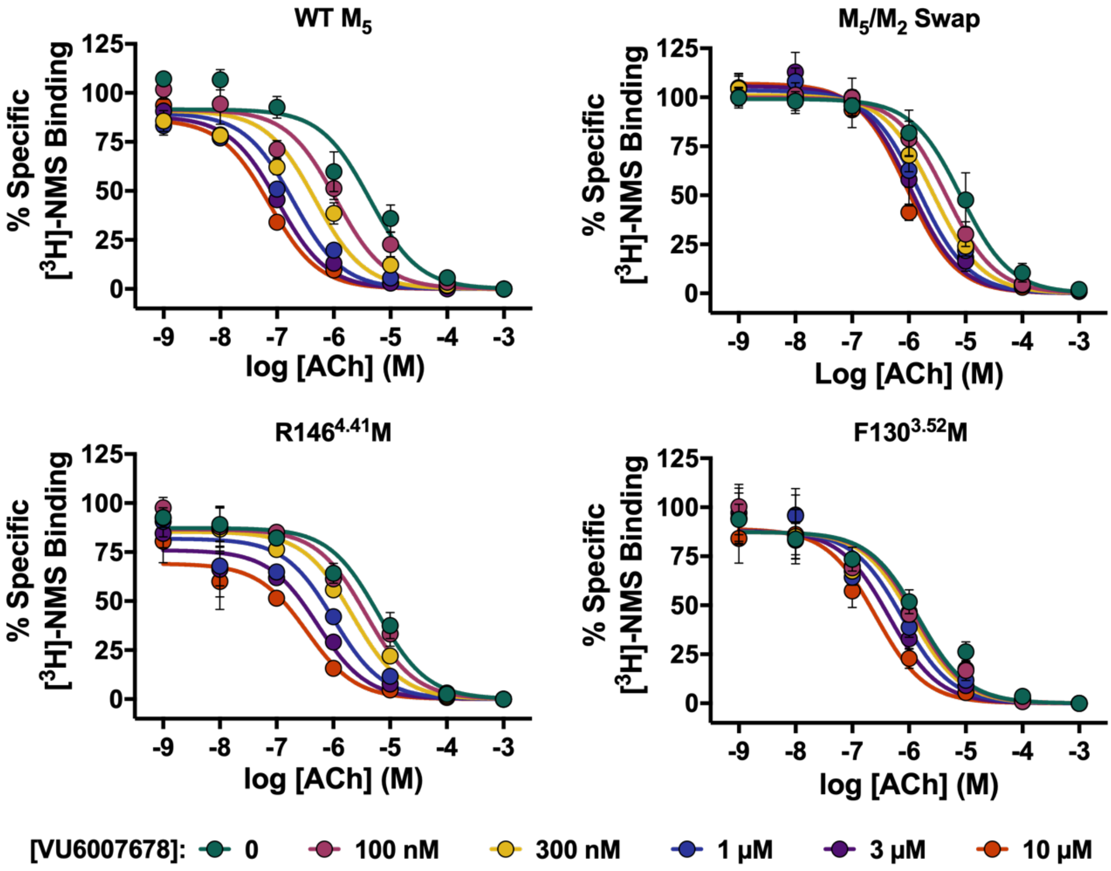

Supplementary Figure 8. Binding interaction of VU6007678 with ACh at the allosteric site mutants. $[^3H]$-NMS equilibrium radioligand binding studies between ACh, VU6007678 and $[^3H]$-NMS. Data points represent mean ± SEM of three to five individual experiments performed in duplicate. WT M_5 mAChR *n* = 3, $F130^{3.52}M$, $R146^{4.41}M$ *n* = 4, M_5/M_2 swap *n* = 5. An allosteric ternary complex model was fit to grouped data. Pharmacology parameters were shared across experiments to yield a single best estimate of each parameter and its associated standard error, as derived from the nonlinear regression algorithm. This global pooled analysis approach ensured model convergence in all instances. Parameters obtained are listed in Supplementary Table 4.

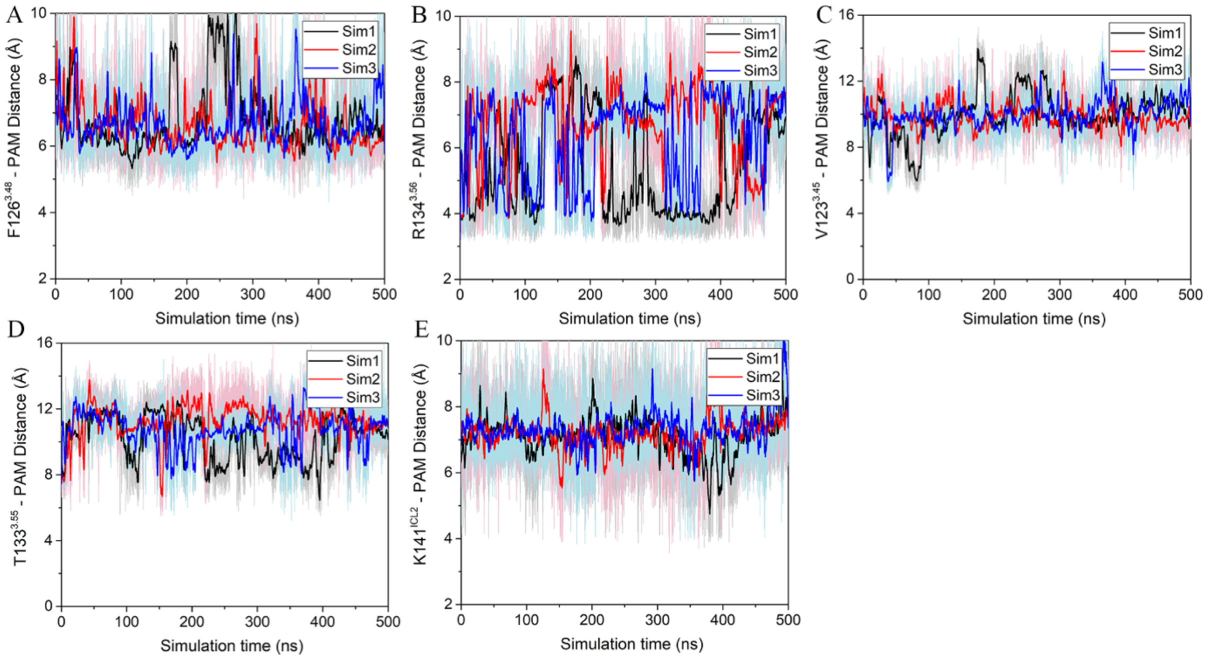

Supplementary Figure 9. Time courses of distances from VU6007678 to allosteric binding site residues in Å calculated from Gaussian accelerated molecular dynamics (GaMD) simulations performed with three separate replicates as indicated through different coloured traces.

Supplementary Table 1 | Functional parameters for the ACh and ML380 interaction in IP1 accumulation assays.

Mutant	IP1 accumulation for the interaction of ML380 vs. ACh			
	ACh pEC_{50}[a]	pK_B[b]	Log τ_B[c]	Log $\alpha\beta$[d]
**WT**	6.94 ± 0.08 (7)	5.43 ± 0.14 (7)	0.32 ± 0.09 (7)	1.48 ± 0.16 (7)
**EH4 Pocket**	6.12 ± 0.11* (3)<br>P < 0.0001	4.80 ± 0.38 (3)	0.64 ± 0.30 (3)	1.74 ± 0.39 (3)
**Y87$^{2.61}$A**	6.40 ± 0.10* (3)<br>P = 0.005	6.34 ± 0.21* (3)<br>P = 0.02	-0.85 ± 0.19* (3)<br>P < .0001	0.83 ± 0.11* (3)<br>P = 0.0008
**Y90$^{2.64}$A**	4.87 ± 0.06* (3)<br>P < 0.0001	5.26 ± 0.11 (3)	-1.35 ± 0.37* (3)<br>P < 0.0001	1.86 ± 0.09 (3)
**I91$^{2.65}$A**	6.79 ± 0.10 (3)	5.73 ± 0.21 (3)	-0.09 ± 0.08 (3)	0.91 ± 0.25* (3)<br>P = 0.004
**E182^{ECL2}A**	6.94 ± 0.09 (3)	5.95 ± 0.24 (3)	0.12 ± 0.07 (3)	0.69 ± 0.33* (3)<br>P < 0.0001
**Q184^{ECL2}A**	6.48 ± 0.13* (3)<br>P = 0.02	5.42 ± 0.19 (3)	0.02 ± 0.11 (3)	1.69 ± 0.22 (3)
**S465$^{6.58}$A**	6.90 ± 0.18 (3)	5.54 ± 0.31 (3)	0.12 ± 0.15 (3)	1.38 ± 0.37 (3)
**D469$^{6.62}$A**	6.71 ± 0.12 (3)	5.59 ± 0.21 (3)	0.15 ± 0.10 (3)	1.64 ± 0.27 (3)
**K470^{ECL3}A**	6.66 ± 0.10 (3)	5.52 ± 0.16 (3)	-0.01 ± 0.08 (3)	1.31 ± 0.19 (3)
**V474$^{7.32}$A**	6.44 ± 0.12* (3)<br>P = 0.01	5.37 ± 0.22 (3)	-0.17 ± 0.10 (3)	1.30 ± 0.22 (3)
**W477$^{7.35}$A**	5.15 ± 0.07* (3)<br>P < 0.0001	5.51 ± 0.12 (3)	-1.14 ± 0.26* (3)<br>P < 0.0001	1.52 ± 0.90 (3)
**H478$^{7.36}$A**	6.42 ± 0.12* (3)<br>P = 0.008	5.68 ± 0.18 (3)	-0.21 ± 0.10 (3)	1.44 ± 0.19 (3)

Data represent the mean ± S.E.M. of (n) independent experiments performed in duplicate. *, significantly different from WT, $p < 0.05$, one-way ANOVA, Dunnett's post hoc test. Statistically significant P values are listed below the mean values.

[a] Negative logarithm of the concentration of ACh required to give half maximal response.

[b] Negative logarithm of the allosteric modulator equilibrium dissociation constant.

[c] Logarithm of operational efficacy parameter.

[d] Logarithm of functional cooperativity between agonist and allosteric modulator.

Supplementary Table 2 | Data collection and refinement statistics

Data Collection	$M_{5\Delta ICL3}$mAChR/mG_{qNi}/scFv16/Iperoxo	$M_{5\Delta ICL3}$mAChR/mG_{qNi}/scFv16/Nb35/ACh/VU6007678
**PDB/EMDB**	9EK0 / EMD-48111	9EJZ / EMD-48110
**Micrographs**	9104	7489
**Electron dose (e^-/Å^2)**	10.57	10.57
**Voltage (kV)**	300	300
**Pixel size (Å)**	0.65	0.82
**Defocus range (μM)**	0.5 - 1.5	0.5 - 1.5
**Symmetry Imposed**	C1	C1
**Particles (final map)**	426,714	418,794
**Resolution (0.143 FSC) (Å)**	2.75	2.06
**Refinement**		
**CCmap_model**	0.84	0.88
**Model Quality**		
**R.m.s deviations**		
**Bond Length (Å)**	0.004	0.005
**Bond angles (°)**	0.722	0.728
**Ramachandran**		
**Favoured (%)**	98.17	98.24
**Outliers (%)**	0	0
**Rotamer outliers (%)**	0.13	0.09
**C-Beta deviations (%)**	0	0
**Clashscore**	4.19	5.15
**MolProbity score**	1.20	1.27

Supplementary Table 3 | Binding parameters for WT M5, M5-M2 TM chimeras

	Saturation Binding with [3H]-NMS		Interaction binding between [3H]-NMS and ACh in the presence of ML380			
	pK_D ([3H]-NMS)[a]	Sites per cell[b]	pK_I (ACh)[c]	Log α (ACh)[d]	pK_B (ML380)[e]	Log α ([3H]-NMS)[f]
**WT M5**	9.12 ± 0.14 (4)	1363757 ± 198445 (4)	5.04 ± 0.07 (3)	0.84 ± 0.18 (3)	5.40 ± 0.20 (3)	-0.47 ± 0.13 (3)
**M5 – M2 TM2, 3, 4**	9.32 ± 0.16 (4)	486397 ± 137869 (4)	4.65 ± 0.08 (3)*, P = 0.001	N.R.	N.R.	N.R.
**M5 – M2 TM3, 4, 5**	9.23 ± 0.27 (3)	1328988 ± 258858 (3)	4.10 ± 0.07 (3)*, P < 0.0001	N.R.	N.R.	N.R.
**M5 – M2 TM1, 7, h8**	8.34 ± 0.03 (4)	443589 ± 53179 (4)	4.75 ± 0.11 (3)*, P = 0.007	2.49 ± 0.55 (3)	3.84 ± 0.51 (3)	=0[g]

Data represent the mean ± S.E.M. of (n) independent experiments performed in duplicate. *, significantly different from WT, one-way ANOVA, Dunnett's post-hoc test, ($P < 0.05$). Statistically significant P values are listed below the mean values. N.D. not determined. N.R. no response.

[a] Negative logarithm of the radioligand ([3H]-NMS) equilibrium dissociation constant.

[b] Number of [3H]-NMS binding sites per cell.

[c] Negative logarithm of the orthosteric agonist (ACh) equilibrium dissociation constant obtained from the allosteric ternary complex model.

[d] Logarithm of affinity cooperativity between the orthosteric agonist (ACh) and allosteric modulator (ML380).

[e] Negative logarithm of the allosteric modulator (ML380) equilibrium dissociation constant.

[f] Logarithm of affinity cooperativity between the orthosteric radioligand ([3H]-NMS) and allosteric modulator (ML380).

[g] Value was not significantly different to zero and such was constrained to this value.

Supplementary Table 4 | Functional and binding parameters for the ACh and VU6007678 interaction in TruPath G protein activation and [^{3}H]-NMS radioligand binding assays.

	TruPath G protein activation				[^{3}H]-NMS radioligand binding				
**Mutant**	**ACh pEC_{50}**[a]	**pK_B**[b]	**log $τ_B$**[c]	**log αβ**[d]	**pK_D**[e]	**Sites per cell**[f]	**pK_B**[g]	**log α (ACh)**[h]	**log α ([^{3}H]-NMS)**[h]
**WT**	5.95 ± 0.09 (8)	4.94 ± 0.24 (8)	0.80 ± 0.21 (8)	2.24 ± 0.27 (8)	9.36 ± 0.08 (5)	587164 ± 66516 (5)	5.67 ± 0.18 (3)	1.83 ± 0.17 (3)	-0.06 ± 0.03 (3)
**Y68$^{2.42}$F**	6.53 ± 0.06 (4)*[1]	4.76 ± 0.28 (4)	1.29 ± 0.28 (4)	2.24 ± 0.30 (4)	9.30 ± 0.09 (4)	641930 ± 103717 (4)	N.D.	N.D.	N.D.
**V123$^{3.45}$I**	5.80 ± 0.12 (4)	5.02 ± 0.26 (4)	0.56 ± 0.22 (4)	2.31 ± 0.29 (4)	9.18 ± 0.09 (3)	1054369 ± 31893 (3)*[7]	N.D.	N.D.	N.D.
**F130$^{3.52}$M**	6.75 ± 0.09 (3)*[2]	N.D.	N.D.	N.D.	9.40 ± 0.13 (4)	782078 ± 98311 (4)	5.07 ± 0.69 (4)	0.99 ± 0.41 (4)	0.03 ± 0.12 (4)
**T133$^{3.55}$A**	6.09 ± 0.11 (3)	5.19 ± 0.21 (3)	1.12 ± 0.20 (3)	2.35 ± 0.25 (3)	9.18 ± 0.05 (4)	419277 ± 63140 (4)	N.D.	N.D.	N.D.
**R134$^{3.56}$A**	6.48 ± 0.09 (3)*[3]	5.08 ± 0.27 (3)	0.97 ± 0.26 (3)	2.11 ± 0.31 (3)	9.27 ± 0.27 (4)	881469 ± 131189 (4)	N.D.	N.D.	N.D.
**R134$^{3.56}$K**	6.20 ± 0.11 (3)	4.98 ± 0.36 (3)	1.06 ± 0.33 (3)	1.78 ± 0.43 (3)	9.44 ± 0.08 (3)	629606 ± 90141 (3)	N.D.	N.D.	N.D.
**K141$^{34.56}$A**	5.64 ± 0.14 (4)	4.75 ± 0.30 (4)	0.62 ± 0.25 (4)	2.34 ± 0.32 (4)	9.28 ± 0.09 (4)	566941 ± 93126 (4)	N.D.	N.D.	N.D.
**R146$^{4.41}$M**	6.83 ± 0.07 (4)*[4]	5.47 ± 0.17 (4)	-0.14 ± 0.11 (4)*[5]	0.57 ± 0.16 (4)*[6]	9.31 ± 0.21 (4)	897976 ± 92116 (4)	5.54 ± 0.23 (4)	1.30 ± 0.20 (4)	-0.23 ± 0.06 (4)
**M_5-M_2 Swap**	5.82 ± 0.13 (3)	N.D.	N.D.	N.D.	9.31 ± 0.16 (4)	466347 ± 98510 (4)	5.98 ± 0.24 (5)	1.02 ± 0.14 (5)	0.07 ± 0.04 (5)

Data represent the mean ± S.E.M. of (n) independent experiments performed in duplicate. *, significantly different from WT, $p < 0.05$, one-way ANOVA, Dunnett's post hoc test. N.D., not determined. Statistically significant P values: *[1]$P = 0.001$; *[2]$P < 0.0001$; *[3]$P = 0.009$; *[4]$P < 0.0001$; *[5]$P = 0.03$; *[6]$P = 0.001$; *[7]$P = 0.01$.

[a] Negative logarithm of the concentration of ACh required to give half maximal response obtained from the three-parameter logistic equation.

[b] Negative logarithm of the allosteric modulator (VU6007678) equilibrium dissociation constant (pK_B) obtained from the operational model of allosterism.

[c] Logarithm of operational efficacy parameter obtained from the operational model of allosterism corrected for receptor expression (sites per cell).

[d] Logarithm of functional cooperativity between agonist (ACh) and allosteric modulator (VU6007678) obtained from the operational model of allosterism.

[e] Negative logarithm of the radioligand ([^{3}H]-NMS) equilibrium dissociation constant.

[f] Number of [^{3}H]-NMS binding sites per cell.

[g] Negative logarithm of the allosteric modulator (VU6007678) equilibrium dissociation constant obtained from the allosteric ternary complex model.

[h] Logarithm of affinity cooperativity between the orthosteric agonist (ACh) or orthosteric radioligand ([^{3}H]-NMS) and allosteric modulator (VU6007678) obtained from the allosteric ternary complex model.
